# Akt Protein Kinase, miR-200/miR-182 Expression and Epithelial-Mesenchymal Transition Proteins in Hibernating Ground Squirrels

**DOI:** 10.3389/fnmol.2018.00022

**Published:** 2018-01-30

**Authors:** Yang-ja Lee, Joshua D. Bernstock, Dace Klimanis, John M. Hallenbeck

**Affiliations:** Clinical Investigation Section, Stroke Branch, National Institute of Neurological Disorders and Stroke, National Institutes of Health (NINDS/NIH), Bethesda, MD, United States

**Keywords:** brain ischemia, cellular reprogramming, epithelial-mesenchymal transition (EMT), hibernation, ischemic tolerance, miR-200 family, miR-182 cluster, Akt (PKB)

## Abstract

Hibernating 13-lined ground squirrels (*Ictidomys tridecemlineatus*; TLGS) rank among the most brain hypoperfusion-tolerant mammals known. Herein we provide some evidence of cycling between an epithelial phenotype and a hybrid epithelial/mesenchymal (E/M) phenotype (partial EMT) within the brains of TLGS during each bout of hibernation torpor. During hibernation torpor, expression of the epithelial marker E-cadherin (E-CDH) was reduced, while expression of the well-known mesenchymal markers vimentin and Sox2 were increased. P-cadherin (P-CDH), which has recently been proposed as a marker of intermediate/partial EMT, also increased during torpor, suggesting that a partial EMT may be taking place during hibernation torpor. Members of the miR-200 family and miR-182 cluster and Akt isoforms (Akt1, Akt2), well-known EMT regulators, were also differentially regulated in the TLGS brain during hibernation bouts. Using SHSY5Y cells, we also demonstrate that the Akt1/Akt2 ratio determined the expression levels of miR-200/miR-182 miRNA family members, and that these miRNAs controlled the expression of EMT-related proteins. Accordingly, we propose that such cell state transitions (EMT/MET) may be one of the mechanisms underlying the extraordinary ischemic tolerance of the TLGS brain during hibernation bouts; hibernator brain cells appear to enter reversible states that confer the stress survival characteristics of cancer cells without the risk of neoplastic transformation.

## Introduction

In the quest to discover effective cytoprotective agent capable of mitigating the damage that occurs during/after an ischemic stroke, our lab has focused on broad, plurifunctional targets that have been shown to maintain homeostasis in hibernation, a state of natural tolerance to brain ischemia. In 13-lined ground squirrels (*Ictidomys tridecemlineatus*; TLGS), cerebral blood flow (CBF) declines to roughly ~10% of baseline rate during hibernation torpor (Frerichs et al., 1994). Such hypoperfusion is equivalent to that which is present in the “ischemic core” of an ischemic stroke, a tissue zone regarded as clinically unsalvageable without the rapid restoration of CBF. Despite this, TLGS show no ischemic damage in the brain upon arousal (Frerichs et al., 1994). Intriguingly, we found a massive 10-fold to 30-fold increase in global small ubiquitin-like modifier (SUMO) conjugation (SUMOylation; involving both SUMO-1 and SUMO-2/3) in the brains of hibernating TLGS (Lee et al., 2007). Global SUMOylation appears to have widespread beneficial effects in the ischemic network, operating in states of tolerance, and acting to preserve homeostasis under stress, and represents a potential “druggable” target (Tempe et al., 2008). Our group and others have shown that increased global SUMOylation is, indeed, a mechanism that provides protection against ischemia in both murine brains and myriad neuronal cell lines (Lee and Hallenbeck, 2013; Bernstock et al., 2018).

It is prudent to note that increased levels of Ubc9 and/or SUMO-conjugation have also been detected in many tumor (cancer) cells (Seeler and Dejean, 2017). These findings raise important questions about hibernators, such as how animals that demonstrate massively increased levels of SUMOylation during hibernation torpor, do not go on to develop tumors. Of note, altered microRNA (miRNA) expression is also frequently seen in human cancers (Calin and Croce, 2006; Croce, 2009) and we have previously shown that the miRNA 183-96-182 cluster and miR-200 family (miR-200a, b, c, miR-141, miR-429) members are consistently down-regulated during hibernation torpor. Such changes are associated with massive increases in the level of SUMOylation and other ubiquitin-like modifiers (ULM) conjugation noted during hibernation torpor (Lee et al., 2012). Critically, these miRNAs have been widely reported to be involved in epithelial-mesenchymal transitions (EMT), wherein cancer cells lose cell-cell contact and maintain “stem-like” properties, both essential criteria for metastasis. The miR-200 family acts as EMT suppressors, while the miR-182 cluster members act primarily as EMT promoters (Pradella et al., 2017). Interestingly, literature has also emerged that stem cells adopt a quiescent state in order to preserve key functional features, paralleling hibernation (itself a quiescent and a highly cytoprotected state; Takeishi and Nakayama, 2016). TLGS undergo multiple cycles of torpor and arousal during the winter months, and they ingest little to no food or water during periods of the arousal (Frerichs et al., 1994; Frerichs and Hallenbeck, 1998). It is curious why these animals would expend the energy needed to periodically rewarm.

Herein, we show that the differential expression of miR-200/miR-182 microRNAs, which is controlled by Akt1/Akt2 isoforms, was associated with EMT-related protein expression in the TLGS brains during hibernation bouts. These results suggest EMT/MET-driven cellular reprograming may happen in the TLGS brains during hibernation.

## Materials and Methods

### Animal Preparation

TLGS were captured by USDA-licensed trappers (TLS Research, Bartlett, IL, USA). This study was carried out in accordance with the recommendations of the NINDS Animal Care and Use Committee (ACUC). The protocol was approved by the NINDS Animal Care and Use Committee. Both male and female TLGS were used equally for this study, and all animals were between 1 and 3 years of age, but because the animals were caught from the wild there is no way to know the exact age of the animals. TLGS were housed individually at 21°C under a 12 h light:12 h dark cycle, and were fed standard rodent diet and water *ad libitum*. To facilitate hibernation torpor, TLGS were transferred to an environmental chamber maintained at 4°C–5°C and 60% humidity and placed separately in cages containing wood shavings (Frerichs et al., 1994). They were kept in darkness, except for a photographic red safe light (3–5 lux). Body temperature (Tb) was measured with an Implantable Programmable Temperature Transponder IPTT-200 (Bio Medic Data Systems). To implant the temperature transmitters, the ground squirrels were first anesthetized with 5% isofluorane and then the transmitter was injected subcutaneously into the middle of the back using a sterile disposable syringe. Animals in six different phases of the hibernation bout (cycle) were differentiated by body temperature (Tb), time and respiratory rate. ACR = active for 4–5 days in the environmental chamber at 4°C–5°C (Tb = 34°C–37°C). ACR represents animals that are capable of entering torpor, but have yet to do so. Ent = entrance phase of hibernation (Tb = 31°C–12°C) after >14 h in the active state since full arousal from a torpor phase of at least 5 days. Torpor phase animals were eligible after they had been in the torpor phase for more than 5 days, aroused and then re-entered the torpor phase. E-hib = early torpor phase (1 day; Tb = 5°C–8°C); L-hib = later torpor phase (>5 days; Tb = 5°C–8°C); AR = arousing from torpor spontaneously with a respiratory rate >60/min and a persistent low body temperature (Tb = 9°C–12°C). IB = interbout aroused from torpor animals are controls that have previously been in the torpor phase of the hibernation bout, but have since returned to normal metabolic conditions in the active state inside the environmental chamber for 7 h (Tb = 34°C–37°C). The 7 h extends from the point that the ground squirrel body temperature has returned to 37°C). We monitored each animal daily. At each time point, brains and other organs (kidney, heart, etc.) were removed quickly, snap-frozen in 2-methylbutane (−50°C), and stored at −70°C until use. At least four animals were used for each time point.

### Micro-RNA Quantification in Brain Samples and in SHSY5Y Cells

Total RNA, including small RNAs, was purified from powdered TLGS brains, kidneys, hearts, or cultured SHSY5Y cells using the mirVana miRNA isolation kit (Invitrogen). Polyadenylation of miRNAs from total RNA and synthesis of first-strand cDNA for use in real-time PCR were carried out using either NCode miRNA First-Strand cDNA Synthesis and qRT-PCR Kits (Invitrogen) or the TaqMan Advanced miRNA cDNA Synthesis Kit (ThermoFisher Scientific). miRNA levels were analyzed by qPCR using the SYBR-Green qPCR (Qiagen)/MyiQ (BioRad) or TaqMan Advanced miRNA assays detection system. For SYBR-Green qPCR, mature sequences (miRbase^1^), of individual miRNAs were used for forward primers and universal primer (from the kit) was used for the reverse primer in each qPCR reaction. In this assay, miRNA expression levels in each sample were normalized to miR-103, one of the most stably expressed (i.e., expression levels were not changed during hibernation bout) miRNAs in squirrel brains (Lee et al., 2012). For TaqMan qPCR, FAM-labeled specific primer sets of each target miRNA were used, and the VIC-tagged primer set for miR-124 (another stably expressed miRNA in squirrel brains) was used for the internal control. Relative expression was calculated using the comparative Ct method (2^−∆∆Ct^; Livak and Schmittgen, 2001).

### Immunoprecipitation and Western Blot Analysis

For tissue extracts for Western blot analyses, frozen whole brains, kidneys, or hearts from TLGS at various phases of hibernation bout were crushed on dry ice to make powder, added to a lysis buffer (2% SDS, 60 mM Tris-HCl (pH 6.8), 50 mM EDTA) with protease inhibitor cocktail (Roche), 2.5 mM sodium pyrophosphate, 1 mM PMSF and 25 mM N-ethylmaleimide, and homogenized on ice. The homogenates were sonicated for 10–15 s heated at 95°C for 10 min and centrifuged at 15,000 *g* for 10 min at 4°C. Total cell extracts from SHSY5Y were made by adding lysis buffer directly to each well on ice. Protein concentrations of the extracts were measured, and the samples were boiled with 5% beta-mercaptoethanol and 2% glycerol before loading of proteins (30 μg/lane) and separated on SDS-PAGE gels (4%–20% in Tris-glycine buffer) along with pre-stained molecular marker standards (SeeBlue Plus 2, Invitrogen) which show molecular weights 250, 148, 98, 64, 50, 36, 22, 16, 6 and 4 kDa. We determined molecular weight of proteins of interest according to the molecular weight standards. Many commercial antibodies did not detect squirrel proteins. When we see positive band(s) with the expected molecular weight, we usually confirm the antibody specificity with human SHSY5Y cell extracts (positive control).

The following antibodies were used for the Western blot analyses: anti-E-CDH (1:500, Abcam), anti-ZO-1 (1:500, Cell Signaling), anti-Zeb1 (1:250, Santa Cruz), anti-P-CDH (1:500, BD Biosciences), anti-Vimentin (1:1000, Abcam), anti-Snai1 (1:500, Cell Signaling), anti-N-Cadherin (anti-N-CDH; 1:500, Santa Cruz), anti-Caveolin (1:1000, Cell Signaling), anti-Sox-2 (1:500, ThermoFisher), anti-β–actin (1:10,000, Sigma). The following antibodies from Cell Signaling Technology were used at 1:1000 dilutions: anti-Akt1, Akt2, p-Akt (S473), p-Akt2 (S-474), p-Akt (T308), GSK3β (total), p-GSK3β (S9), PRAS40 (total) and p-PRAS40 (T246). Intensities of bands were analyzed using the densitometric functions of ImageJ (NIH). The intensities of target proteins were normalized with the intensities of β-actin bands on the same membrane. For immunoprecipitation (IP), frozen crushed brain tissue was homogenized in buffer A (10 mM Tris, pH 8, 1 mM EDTA, pH 8, 0.1 mM MgCl_2_, 100 mM NaCl and 1% Triton x-100) containing protease/phosphatase inhibitor cocktail (Sigma, MS-SAFE), and IP was performed with Dynabeads/protein G (Life Technology) according to the manufacturer’s instruction. Anti-Ago2 (clone 11A9, Millipore), anti-GMP-1 (Life Technology), or anti-SUMO-1 (in house) were used for IP.

### Transfection of miRNA Mimics and/or Inhibitors, AKT siRNAs and Mammalian Expression Constructs

Human neuroblastoma SHSY5Y cells were transfected with miRNAs (miR200c, miR183, miR182, miR141, miR-96, miR-34) mimics or inhibitors along with negative controls (Thermo Scientific Dhamacon miRIDIAN microRNA Mimics and Hairpin Inhibitors) using Lipofectamin RNAimax (Invitrogen). 40–120 nM (final concentration) of mimics or inhibitors were used in each transfection. SHSY5Y cells were also transfected with siRNAs (Trilencer-27 siRNA; Origene Tecnology) specific to either Akt1 or Akt2 with or without Akt1 or Akt2 mammalian expression construct (cloned in pCMV6-AN-Myc-DDKvector; Origene Technology) by Lipofectamine RNAimax (Invitrogen). In each transfection, siRNA at 40 nM and DNA constructs at 0.5 μg were used with 3 × 10^5^ cells per well (six well plate). Scrambled siRNA and empty vector were used as controls. Typically, 3 days after each transfection, microRNA levels (by qPCR) and/or protein levels (by Western blot) were checked.

### Statistical Analysis

To test for differences in various phases of hibernation bout vs. control (i.e., ACR), a one-way ANOVA followed by Dunnett’s *post hoc* test was performed. Values of *p* ≤ 0.05 were deemed to be significant.

## Results

### Expression of miR-200 and miR-182 Families Is Tightly Controlled (Differentially Expressed) in the Brain of TLGS during Hibernation Bouts

**Figure 1:**
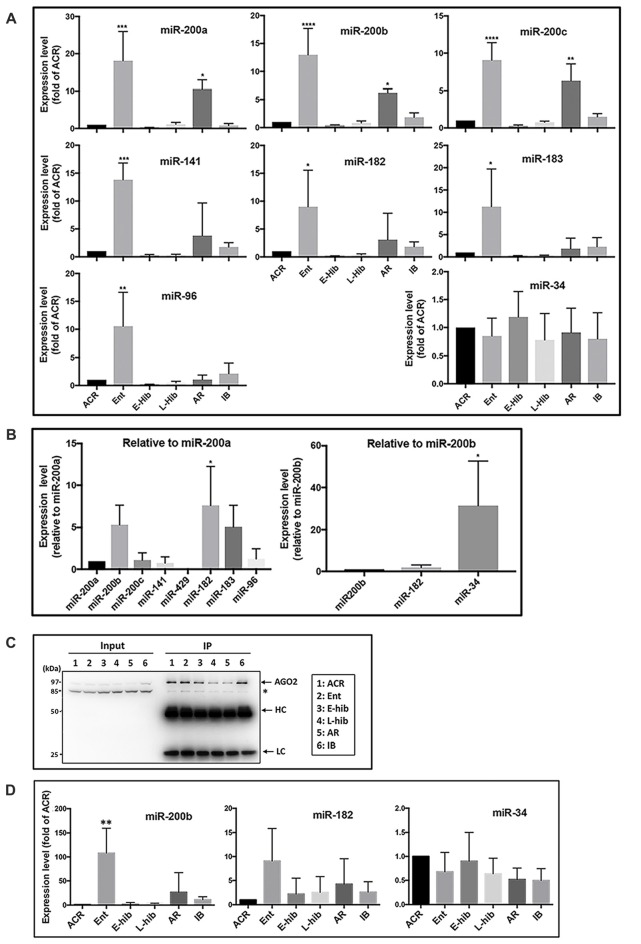
Expressions of miR-200 family and miR-182 cluster members of miRNA are differentially regulated in squirrel brains during hibernation bouts. **(A)** Expression levels of miR-200 family (miR-200a, b, c and miR-141) and miR-182 cluster (miR-182, miR-183 and miR-96) members in addition to miR-34 in squirrel brains at different stages of hibernation bout. The levels of these miRNAs were measured by qPCR, normalized (∆Ct) by the level of miR-124 and/or miR-103 (i.e., stably expressed miRNAs that did not change during hibernation bout), compared to the expression of ACR (∆∆Ct) and showed as fold-changes of ACR (2^−∆∆Ct^). **(B)** Left: comparisons of expression levels of miR-200 family and miR-182 cluster members of miRNAs in squirrel brains from the active phase (ACR), shown as folds vs. miR-200a. Right: comparison of expression level of miR-34 to miR-200/miR-182 family members in the squirrels’ brain from ACR, shown as folds vs. miR-200b. **(C)** Representative immunoblots of AGO2-immunoprecipitants of squirrel brain samples from various hibernation stages. HC: IgG heavy chain; LC: IgG light chain. **(D)** miRNA (miR-200b, miR-182 and miR-34) levels in the AGO2-immunoprecipitants of brain samples from various stages of hibernation bout. They were measured by qPCR, normalized (ΔCt) by the level of miR-124, compared to the expression of ACR (∆∆Ct), and showed as fold-changes of ACR (2^−∆∆Ct^) as shown in **(A)**. ACR, active in cold room; Ent, entrance; E-hib, early torpor; L-hib, late torpor; AR, arousal; IB, interbout. Data represent the mean ± SD of at least four different brain samples from each stage of hibernation bout. The statistical analyses were shown relative to the ACR group. *p < 0.05, **p < 0.01, ***p < 0.001, ****p < 0.0001.

MicroRNA microarray analyses of TLGS brains (obtained during active and late torpor phases) showed that the miR-200 family (miR-200a, b, c/miR-141/miR-429) and the miR-182 cluster (miR-182/miR-183/miR-96) members were among the most consistently depressed miRNAs in the brains of animals in torpor compared to active animals (Lee et al., 2012). Following that, we examined miRNA levels at all phases of hibernation, including active in cold room (ACR), entrance (Ent), early torpor (E-hib), late torpor (L-hib), arousal (AR) and interbout (IB) phases. Concordant with our previous report (Lee et al., 2012), expressions of these miR-200 family and miR-182 cluster members were reduced (as low as 10% of active phase) in the brains of hibernating TLGS during torpor phases (E-hib and L-hib; Figure 1A). Interestingly, levels of these microRNAs were massively increased (>10-fold vs. active phase) during the entrance phase, with the difference in expression between Ent (highest) and E-hib (lowest) phases reaching 100-fold (Figure 1A). Post-torpor, expression of these microRNAs was restored and increased during the arousal phase (second highest levels during hibernation bout), before decreasing to active phase levels at IB (Figure 1A).

As such, expression of miR-200 family and miR-182 cluster miRNAs was regulated in a similar manner during the hibernation bout. As mentioned previously, the miR-200 family comprises five miRNAs (miR-200a, b and c; miR-141; and miR-429) and the miR-182 cluster contains miR-182, miR-183, and miR-96. The relative expression levels of these miRNAs in the ACR brains are shown in the Figure 1B (left panel). Among miR-200 family members, miR-200b demonstrated the highest level of expression, followed by miR-200c, miR-200a and miR-141, with miR-429 expression undetectable. MiR-182 and miR-183 were expressed at similar levels as miR-200b, but miR-96 was expressed as a much lower level.

The miR-200 family and miR-182 cluster are well-known regulators of EMT (Zhang et al., 2015; Senfter et al., 2016). On the other hand, the expression of the miR-34 family miRNAs, which are also known to be major regulators of EMT (Hahn et al., 2013), were not changed during the hibernation bout (Figure 1A). In addition, the expression levels of miR-103 and miR-124, which we used as internal controls, did not change throughout hibernation bout. Collectively, these data suggest that the differential expression of miR-200 family and miR-182 cluster members are not universal but rather represent a unique phenomenon that occurs during the hibernation bout. The expression level of miR-34 in the active squirrel brain was, however, significantly higher than any of the miR-200/miR-182 members (Figure 1B, right panel). Recently, Salzman et al. (2016) reported that a pool of mature but inactive miR-34 existed within the cell that can be readily activated upon DNA-damage via 5′-end phosphorylation. Accordingly, we further interrogated the miR-34 detected during the hibernation bout in an effort to differentiate inactive and active forms via the IP of Ago (Elkayam et al., 2012). We immunoprecipitated TLGS brain extracts from different phases of hibernation bout with an anti-Ago2 antibody (Figure 1C), and went on to measure the amounts of miR-34, miR-200b and miR-182 that were pulled down. As shown in Figure 1D, the amount of active miR-34 did not significantly differ throughout hibernation bout, reflective of trend noted for total miR-34 levels (Figure 1A). Levels of active miR-200b or miR-182 were still differentially regulated as per the total amount (Figures 1A,D), even more profoundly in the case of miR-200b (Figure 1D).

### Partial (Reversible) EMT Occurs in TLGS Brains during Hibernation Bouts

**Figure 2:**
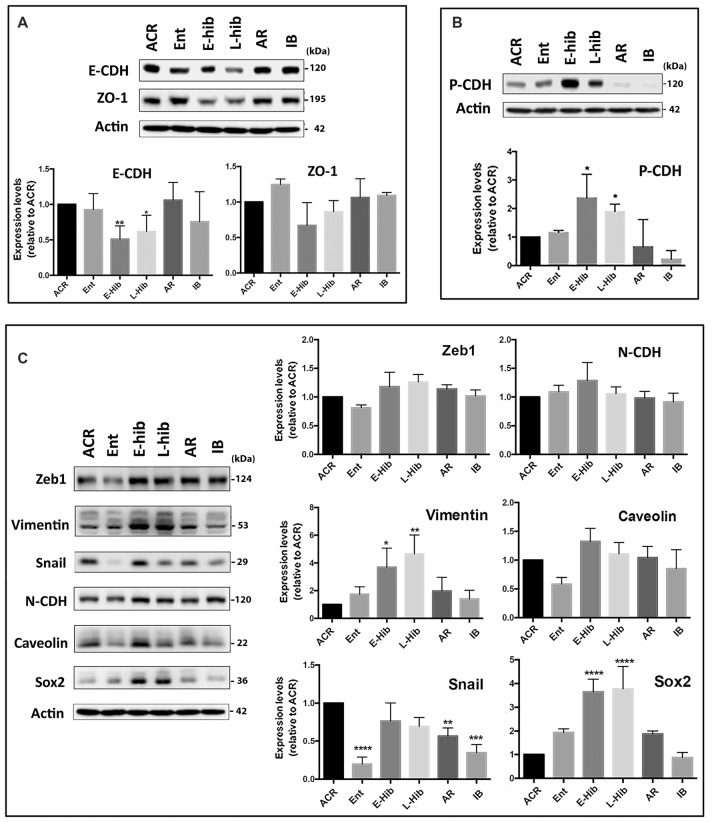
Expression levels of epithelial-mesenchymal transition (EMT)-related proteins in squirrel brains during hibernation bouts. **(A)** Representative immunoblots of epithelial markers E-cadherin (E-CDH) and ZO-1 in brain extracts from various stages of hibernation (upper panel), and their quantitative analyses (lower panel). **(B)** An intermediate (partial) EMT marker, P-cadherin (P-CDH), was differentially expressed in squirrel brains during hibernation bouts. Representative immunoblot (upper) and its quantitation (lower). **(C)** Representative immunoblots of several mesenchymal markers in the brain extracts from various stages of hibernation bout (upper) and their quantitative analyses (lower). ACR, active in cold room; Ent, entrance; E-hib, early torpor; L-hib, late torpor; AR, arousal; IB, interbout. Data represent the mean ± SD of at least four different brain samples from each stage of hibernation bout. The statistical analyses were shown relative to the ACR group. *p < 0.05, **p < 0.01, ***p < 0.001, ****p < 0.0001.

Being that the miR-200 family and miR-182 family are well known regulators of EMT (Zhang et al., 2015; Senfter et al., 2016), we examined the expression of EMT-related proteins in TLGS brain extracts at various stages of the hibernation bout. Being that no commercial antibodies are made using squirrel proteins as antigens, we utilized antibodies raised against human, mouse, or rat proteins; accordingly, we attempted to use several different antibodies targeting different epitopes and/or species in our protein immunoblots. In so doing, we found several EMT-related proteins that were differentially expressed in squirrel brains during the hibernation bout. Expression of an epithelial marker, E-cadherin (E-CDH), was reduced during hibernation torpor (E-hib and L-hib; Figure 2A). Meanwhile, expression of a well-known mesenchymal marker, vimentin, was increased significantly during hibernation torpor (Figure 2C). Moreover, expression of Sox2, a transcription factor with a critical role in stem cell maintenance and often used as a mesenchymal marker, increased significantly during hibernation torpor as well (Figure 2C). Among other commonly employed mesenchymal markers, Snail displayed an interesting pattern: the expression was depressed in the entrance phase (Ent), increased during torpor phases, then decreased to the active phase (ACR) level (Figure 2C). Similar patterns were seen when zinc finger E-box-binding homeobox 1 (Zeb1) and Caveolin were examined yet these failed to reach statistical significance (Figure 2C). Of note, we did not detect any significant changes in the expression of N-CDH, one of the most referred mesenchymal markers, in TLGS brains during hibernation bout (Figure 2C). We did however reveal a remarkable differential expression of P-CDH, which has recently been proposed as a marker of intermediate/partial EMT (Ribeiro and Paredes, 2014). As shown in Figure 2B, P-CDH was expressed at relatively low levels during active (ACR, IB) or transition (Ent, AR) phases, but increased significantly during torpor (E-hib and L-hib) phases, which suggests that a partial/intermediate EMT is taking place during hibernation torpor, and is reversed during arousal-IB phases. Interestingly, this partial EMT occurs not only in TLGS brains, but also in their hearts during hibernation torpor (Supplementary Figure S1).

### MiR-200 Family and miR-182 Cluster Members of miRNAs Are Able to Directly Control the Expression of Proteins Involved in the EMT/MET Process in SHSY5Y Cells

**Figure 3:**
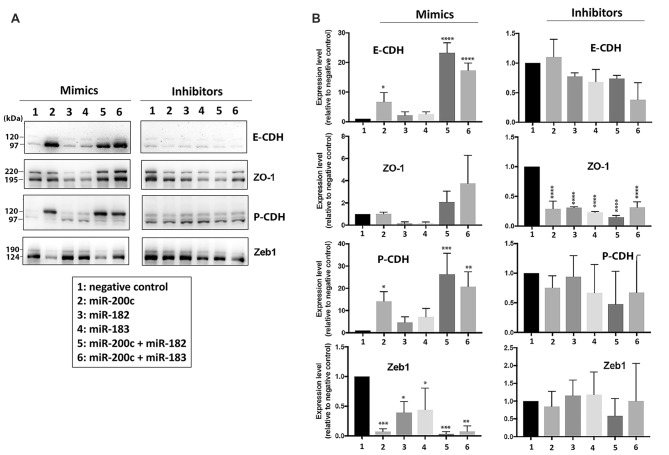
Effect of overexpression or depletion of miRNAs on the expression of EMT-related proteins in SHSY5Y cells. **(A)** Representative immunoblots of EMT-related proteins. Left, overexpression by mimics; Right, depletion by inhibitors. **(B)** Quantitative analyses of EMT-related protein expressions. Left, overexpression of miRNA by mimics, Right, depletion of miRNA by inhibitors. 1, negative control; 2, miR-200c; 3, miR-182; 4, miR-183; 5, miR-200c + miR-182; 6, miR-200c + miR-183. Data represent the mean ± SD of at least three independent experiments. The statistical analyses were shown relative to the negative control group. *p < 0.05, **p < 0.01, ***p < 0.001, ****p < 0.0001.

A human neuroblastoma cell line, SHSY5Y, was transfected either with mimics or inhibitors of miR-200 family or miR-182 cluster miRNAs to investigate if these miRNAs could affect the expression levels of EMT-related proteins. As shown in Figures 3A,B (left panels), overexpression of miR-200c increased the expression of E-CDH (an epithelial marker) and P-CDH (an intermediate marker) greatly, but significantly decreased the expression of the mesenchymal marker Zeb1. The effects of overexpression of miR-182 or miR-183 alone were minimal but in combination with miR-200c were profound. Transfecting inhibitors of these microRNAs resulting in minimal effects (Figures 3A,B, right panels). Being that SHSY5Y cells were tumor cell lines (very low E-CDH and high Zeb1, see Figure 3A lane 1), and the endogenous levels of these microRNAs were already very low (unpublished data), it is unsurprising that inhibitors had minimal impact. Interestingly, P-CDH was regulated the same way as epithelial markers in this cell system, which might have come from using tumor cell lines (already a mesenchymal phenotype) as well.

### Akt Isoforms (Akt1 and Akt2) Are Differentially Expressed in the Brain of TLGS during Hibernation Bout

**Figure 4:**
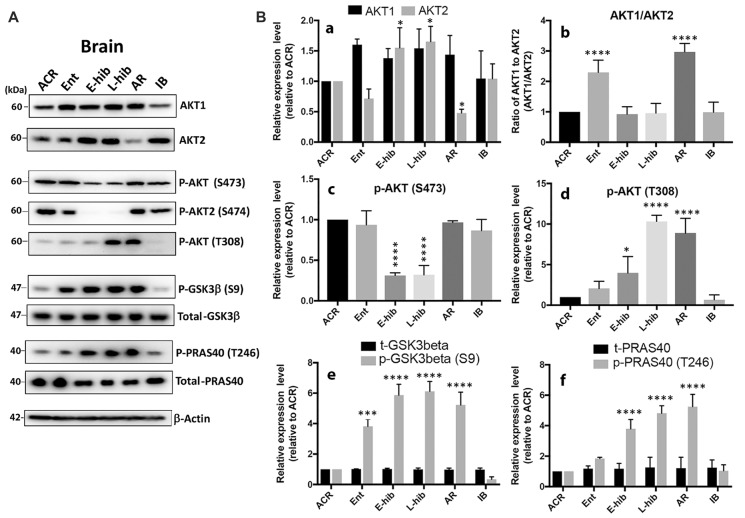
Akt isoforms (Akt1 and Akt2) are differentially expressed in the brain of 13-lined ground squirrels during hibernation bout. **(A)** Representative immunoblots of Akt isoforms, Akt substrates (GSK3β, PRAS40), and their phosphorylated forms in the squirrel brain during hibernation bout. **(B)** Quantitative analyses of these protein expressions. **(a)** Akt1 and Akt2; **(b)** the ratio of Akt1/Akt2; **(c)** p-Akt (S473); **(d)** p-Akt (T308); **(e)** total- and phosphor- GSK3β (t- and p-GSK3β); **(f)** total and phosphor-PRAS40 (t- and p-PRAS40). ACR, active in cold room; Ent, entrance; E-hib, early torpor; L-hib, late torpor; AR, arousal; IB, interbout. Quantitation data represent the mean ± SD of at least four different brain samples from each stage of hibernation bout. The statistical analyses were shown relative to the ACR group. *p < 0.05, ***p < 0.001, ****p < 0.0001.

Naturally, we wondered how these miRNAs were regulated in TLGS brains during hibernation bouts. Iliopoulos et al. (2009) reported that the ratio of Akt1/Akt2 determines the abundance of miR-200 miRNA family members: a high Akt1/Akt2 ratio increases their abundance, and a low ratio reduces their abundance. Thus, we examined whether Akt isoforms were differentially expressed in TLGS brains during hibernation bouts. As shown in Figures 4A,Ba, Akt1 levels slightly increased, although this was statistically non-significant, upon entering hibernation, but returned to the original level after waking up (IB), while Akt2 levels increased significantly during torpor phases (E-hib and L-hib) but decreased slightly during entrance (Ent) and significantly at arousal (AR) phases. When we plotted the ratio of Akt1/Akt2 (Figure 4Bb), we saw significant increases during entrance and arousal phases, when the expression of miR-200 family microRNAs was greatly increased (Figure 1A). Interestingly, during hibernation torpor (E-hib, L-hib), the phosphorylation of Akt at S473 (S474 for Akt2), which is supposed to be essential for full Akt activity, was significantly reduced (Figures 4A,Bc) even though phosphorylation at another essential site for Akt activity (T308) was increased (Figure 4Bd). We wondered whether Akt, which was phosphorylated at T308 but not phophorylated at S473 (S474 for Akt2) during hibernation torpor, was active or not. GSK3β and PRAS40 are well known Akt substrates (Vivanco et al., 2014). We checked the phosphorylation status of Akt phosphorylation sites of these proteins in TLGS brains at different stages of hibernation bout. Phosphorylation of both GSK3β and PRAS40 were increased during hibernation torpor despite the absence of S473 phosphorylation of Akt (Figures 4A,Be,f), suggesting that the phosphorylation at S473 is not necessary for Akt activity in hibernating TLGS. The differential expression of Akt isoforms and altered phosphorylation patterns observed in TLGS brains during hibernation bout was also seen in TLGS kidneys (Supplementary Figure S2).

### High Akt1/Akt2 Expression Ratios Contribute to High Abundancy of miR-200 Family and miR-182 Cluster Members of miRNAs in SHSY5Y Cells

**Figure 5:**
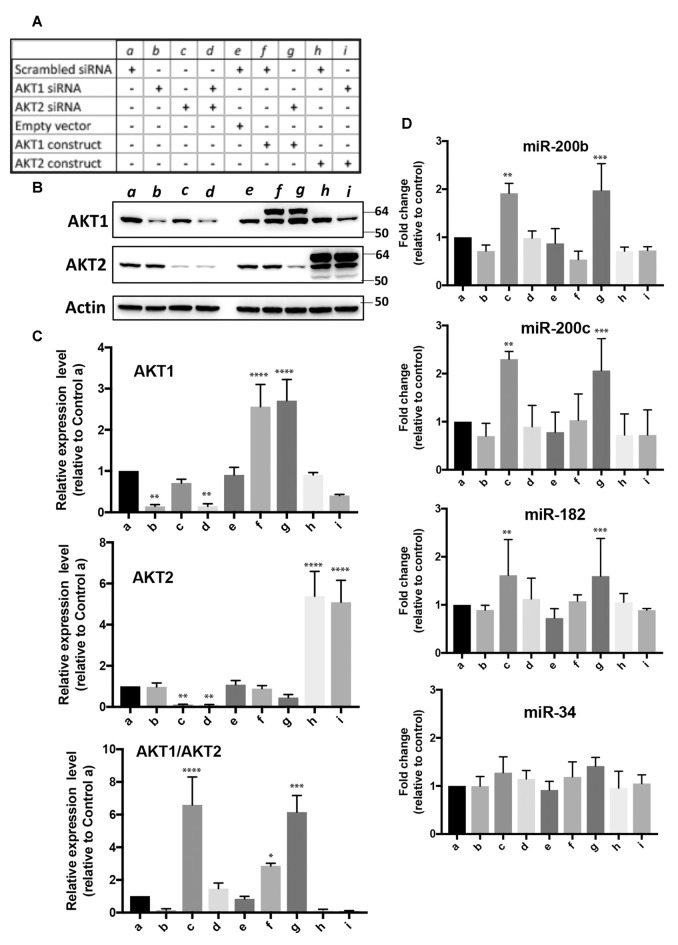
Effect of over-expression and/or depletion of Akt1 and/or Akt2 on the expression of miR-200b, c, miR-182 and miR-34 in SHSY5Y cells. **(A)** Nine conditions (a–i) for overexpression or depletion of Akt isoforms used in this experiment. **(B)** Representative immunoblots of Akt1 and Akt2 proteins under these nine conditions. **(C)** Quantitative analyses of Akt1 and/or Akt2 protein expressions, and the ratio of Akt1/Akt2 expressions. **(D)** Expressions of miR-200b, c, miR-182 and miR-34 under these nine conditions. Quantitation data represent the mean ± SD of at least three independent experiments. The statistical analyses were shown relative to the control group a. *p < 0.05, **p < 0.01, ***p < 0.001, ****p < 0.0001.

In order to investigate the effects of depletion or overexpression of Akt1 and/or Akt2 on miRNA expression, we transfected SHSY5Y cells with siRNAs specific to each Akt isoform ± Akt 1 or Akt2 construct in mammalian expression vector in nine combinations (*a–i*) shown in Figure 5A. The Akt1 and Akt2 proteins were depleted or overexpressed accordingly (Figures 5B,C). The Akt1/Akt2 protein expression ratio in each condition was plotted as well (Figure 5C, bottom). The ratio was very high in conditions *c* and *g*, i.e., Akt2 siRNA with or without Akt1 overexpression (Figure 5C, bottom). Under these conditions, we measured the expression levels of miR-200b, miR-200c, miR-182 and miR-34. As shown in Figure 5D, the expressions of miR-200b, miR-200c and miR-182 were increased only in condition *c* or *g* where the Akt1/Akt2 ratio was high. These results show that the Akt1/Akt2 ratio indeed affects the expression of miR-200 family and miR-182 cluster members, even though the effects were not very strong (<3-fold). Interestingly, miR-34 expression was not affected at all in any conditions (Figure 5D, bottom).

## Discussion

Hibernation is an excellent model of natural tolerance to ischemia. Mammalian hibernators undergo a remarkable phenotypic switch that involves profound changes in physiology, morphology, and behavior in response to periods of unfavorable environmental conditions (Drew et al., 2001; Carey et al., 2003; Storey, 2003). During torpor, hibernating animals lower their energy consumption, blood flow and body temperature to otherwise lethal levels, but because of special adaptive changes, suffer no CNS damage or cellular loss (Heller, 1979; Frerichs and Hallenbeck, 1998; Zhou et al., 2001; Dave et al., 2012; Lee and Hallenbeck, 2013; Tissier et al., 2014). Interestingly, isolated brain tissue (organotypic slices) from active animals of species capable of hibernation (TLGS, AGS), were more tolerant to hypoxia, hypothermia and/or aglycemia than slices from species unable to hibernate (e.g., rats; Pakhotin et al., 1990; Frerichs and Hallenbeck, 1998; Dave et al., 2006; Christian et al., 2008; Bogren and Drew, 2014), thereby suggesting that hibernators have innate systems/strategies that allow for tolerance to these stresses.

We found that expression of the miR-200 family and the miR-182 cluster members of miRNAs in TLGS brain was tightly controlled during hibernation bouts (Figure 1). These miRNAs were initially found to be involved in the regulation of TLGS brain SUMOylation during periods of hibernation torpor (Lee et al., 2012). Interestingly, these miRNAs were also well-known as regulators for EMT (Mutlu et al., 2016; Senfter et al., 2016). EMT is an essential process by which cells lose their epithelial phenotype and acquire mesenchymal properties, which include a decreased expression of epithelial markers, such as E-CDH, and an increased expression of mesenchymal markers, such as N-CDH, fibronectin, and vimentin (Singh et al., 2017). Cyclic transition from the epithelial state to a partial EMT state plays a crucial role in organogenesis, wound healing, and tissue regeneration, but can be “hijacked” for cancer cell metastasis (Jolly et al., 2015).

Since one miRNA can target hundreds of mRNAs, each miRNA regulates many different biological functions (Takahashi et al., 2014). However, most of the reported functions of miR-200 family members have been EMT-related (Senfter et al., 2016). The miR-200 family plays an essential role in EMT suppression, mainly through targeting Zeb1/2. These transcription factors bind to the promotor of E-CDH and silence its expression (Vannier et al., 2013), which can lead to EMT. Also, Zeb1/2 and miR-200 are involved in a double-negative feedback loop in which the miR-200 family members target and suppress Zeb1/2 and promote epithelial differentiation (Hill et al., 2013). Conversely, Zeb1 expression controls miR-200 expression through Zeb-binding sites in the common promoter region of the miR-200 family (Hill et al., 2013), thus inducing a mesenchymal pattern of gene expression. As for the miR-182/96/183 cluster, its role in tumor biology remain controversial. While most studies suggest that the miR-182 cluster has pro-tumorigenic and pro-metastatic effects in cancers (Lei et al., 2014; Li et al., 2014; Yang et al., 2014; Leung et al., 2015; Yu et al., 2016), other groups have reported an inhibition of these effects (Zhang et al., 2011; Wang et al., 2016). This discrepancy might arise from the special role of miR-182 (and its cluster members) in EMT/metastasis. Cancer cell metastasis involves a multi-step progression: tumor cells are disseminated from primary tumors by EMT that starts the metastatic process. Then, after dissemination, these cells undergo a controlled cell state reversal from EMT to MET, in order to locally colonize and form macrometastases within distant organs, thus completing the metastatic cycle (Tsai and Yang, 2013). Zhan et al. (2017) reported recently that, unlike miR-200 family members that are involved in the initial steps of EMT, miR-182 activates MET, the reversal of EMT, by targeting a miR-182 suppressor, SNAI1 (Snail; an EMT marker), and higher levels of miR-182 were detected in metastatic tissues compared with paired primary tissues, both of which are epithelial-like states. As such, miR-182 (and its cluster members) are pro-tumorigenic and pro-metastatic since they are detected at higher levels in the metastatic tissues, but they are EMT inhibitors since they function to reverse EMT. It is intriguing that the expression patterns of miR-200 family and miR-182 cluster members were similar in TLGS brain during hibernation bouts (Figure 1). Are these mediators of cell state change actively counterpoised in hibernation torpor to prevent cell state transition from progressing to full EMT, or do these two different families of miRNAs have similar functions in hibernating animals? In any case, phenotypes of these miRNAs should be the same: high expression promotes an epithelial state and low expression a mesenchymal state.

How do these miRNAs control EMT and/or MET? They would be able to directly control the expression of proteins involved in the EMT/MET process. For example, overexpression of miR-200 repressed expression of a mesenchymal marker, Zeb1, and increased expressions of the epithelial markers, E-CDH and ZO-1, in a neuroblastoma cell line, SHSY5Y (Figure 3). Overexpression of miR-182/183 alone had a small effect (or negative effect on ZO-1), but together with miR-200 enhanced E-CDH and ZO-1 expressions (Figure 3). Accordingly, miRNAs could control EMT/MET directly in the squirrel brain during a hibernation bout. As we mentioned above, massive global SUMOylation occurs in the squirrel brain during hibernation torpor (Lee et al., 2007), and repression of miR-200 family and miR-182 cluster members upregulate, at least partially if not completely, this important post-translational modification (Lee et al., 2012). Regulation of EMT through SUMOylation of transcription factors has been described (Bogachek et al., 2015). MiR-200 family and miR-182 cluster members, therefore, could have a strong influence on the EMT/MET process through controlling SUMOylation levels in TLGS brain during the hibernation cycle. SUMOylation of transcription factors or EMT-related proteins generally leads to EMT inhibition (Wang et al., 2010; Ren et al., 2014; Chandhoke et al., 2016; Zhang et al., 2016), which fits with our proposal that the progression of EMT is reversed (or inhibited) at the end of hibernation torpor where SUMOylation returns to basal levels. Unfortunately, we could not yet pinpoint any transcription factors whose SUMOylation levels increased during hibernation torpor.

We wondered how these miRNAs’ expressions were regulated in TLGS brains during hibernation bouts. Iliopoulos et al. (2009), by using spontaneously immortalized *Akt^−/−^/Akt2^−/−^/Akt3^−/−^* mouse lung fibroblasts each expressing an individual Akt isoform, found that induction of EMT is controlled by miR-200 the abundance of which depends on the balance between Akt1 and Akt2 rather than on the overall activity of Akt. They suggested that a shift in the relative abundance or activity of Akt1 and Akt2 will alter the abundance of members of the miR-200 miRNA family and consequently the invasiveness and oncogenic potential of human tumors. The ratio of Akt1/Akt2 determines the abundance of miR-200 miRNA family members: a high Akt1/Akt2 ratio increases their abundance (more epithelial phenotype), and a low ratio reduces their abundance (more mesenchymal phenotype). Indeed, we found that the Akt1/Akt2 expression ratio was significantly increased in Ent and AR phases of hibernation bout both in the brain (Figure 4) and the kidney of TLGS (Supplementary Figure S2), when the expression of miR-200 family miRNAs were greatly increased (Figure 1A). We believe that differential expression of Akt isoforms contributes, at least in part, to the differential expression of miR-200 family members of miRNAs in hibernating TLGS. We show here that differential expression of Akt isoforms leads to differential expression of the miR-200 family, which appears to regulate EMT. However, it remains unclear what triggers (or causes) the differential expression of Akt isoforms. Akt is the primary downstream mediator of PI3K signaling which is activated by various growth factors (Brazil and Hemmings, 2001; Nicholson and Anderson, 2002). How Akt isoforms are expressed/activated in the nutrient deficient environment of hibernation may be an interesting direction for future research. Low temperature and hypoxia, as well as deprivation of nutrients including glucose (a central aspect of hibernation) may all be involved.

Akt is a serine-threonine protein kinase that influences numerous cellular processes including survival, proliferation, metabolism and migration (Song et al., 2005; Manning and Cantley, 2007). Most of these functions depend on its kinase activity, which is activated by phosphorylation at two sites (T308 and S473). Although 3′-phosphoinositide-dependent kinase 1 (PDK1) has been identified as the T308 kinase, the identity of the S473 kinase remains controversial (Toker and Newton, 2000; Persad et al., 2001; Jacinto et al., 2006; Islam et al., 2014). Akt supposedly must be phosphorylated both at T308 and S473 to be fully activated, and oftentimes S473 phosphorylation alone is used for demonstrating Akt activity. Interestingly, Akt S473 is known to be hypo-phosphorylated in hibernating TLGS during torpor (Cai et al., 2004; Abnous et al., 2008; Wu and Storey, 2012), and Akt activity is considered to be repressed during torpor phase. However, we show here evidence of Akt activity, despite S473 hypo-phosphorylation, by way of phosphorylation of its substrates, GSK3β and PRAS40, during torpor phase in TLGS (Figure 4). Indeed, one other report found that Akt activity was independent of S473 phosphorylation (Hill et al., 2001). Akt activity independent of S473 phosphorylation seems rare, but in the future may be found more often using careful study design. The Akt family of kinases includes three members (Akt1/Akt2/Akt3) that share a high degree of homology. Akt1 and Akt2 are broadly expressed in most tissues, where Akt3 has a more limited expression pattern (Yang et al., 2003). All Akt isoforms are involved in the induction and progression of human cancer (Altomare and Testa, 2005), but the Akt1 and Akt2 isoforms play different roles in certain cancers (Hutchinson et al., 2004; Irie et al., 2005; Maroulakou et al., 2007; Dillon et al., 2009): Akt1 is critical for cancer induction and Akt2 for metastatic dissemination. Iliopoulos et al. (2009) identified molecular details regarding the opposing functions of the highly homologous Akt isoforms in cell migration: the balance (ratio) between Akt1 and Akt2 regulates the abundance of miR-200, which in turn controls the induction of EMT. Until now, there have been no other reports which support their findings. Here, we reproduced their findings in a human neuroblastoma cell line, SHSY5Y. It is very interesting that the correlations were also seen in non-cancerous TLGS brains (and kidneys) under the physiological conditions (hibernation).

From our previous work (Lee et al., 2012) we know that manipulating miR-200/miR-182 families protects rat neurons and SHSY5Y cells from OGD stress. Now, based on the results from TLGS brain tissue, we have confirmed in the human neuroblastoma cell line SHSY5Y that the expressions of the miR-200 and miR-182 clusters are regulated by the Akt1/Akt2 ratio, and furthermore that overexpression or depletion of these miRNAs caused differential expression of EMT-related proteins. All these results suggest that hibernating brain cells may enter reversible states that confer stress survival characteristics of cancer cells without progression to neoplastic transformation. The regulation of plasticity and bi-directional conversion in cell states (EMT/MET) may underlie the robust protection against the severe brain hypoperfusion observed during TLGS hibernation. It would be of considerable interest to identify (or dissect) the cell types that are involved in the EMT/MET transitions we observed in hibernating TLGS brain. Unfortunately, it was not possible in our current system, which was one of the limitations of our study. Though the Akt1/Akt2 ratio was found to regulate expression of miR-200 and miR-182 in a neuroblastoma cell line, and modulation of these miRNAs caused differential expression of EMT-related proteins, whether this is the case in hibernating TLGS remains inconclusive. For a more definitive conclusion, we should use neurons or NPC isolated from TLGS as has been described (Drew et al., 2011, 2016) for the future work.

## Author Contributions

YL designed, performed experiments, analyzed data and wrote the manuscript. JDB designed experiments and wrote the manuscript. DK prepared all hibernator samples, and JMH supervised the whole research. All authors have reviewed and approved the manuscript.

## Conflict of Interest Statement

The authors declare that the research was conducted in the absence of any commercial or financial relationships that could be construed as a potential conflict of interest.

## Footnotes

^1^http://www.mirbase.org/ftp.shtml

## Supplementary Material

FIGURE S1 Expression levels of EMT-related proteins in the squirrels’ kidney and heart during hibernation bouts. **(A)** Representative immunoblots of EMT markers in the kidney extracts from various stages of hibernation bouts (upper panel), and their quantitative analyses (lower panel). **(B)** Representative immunoblots of EMT markers in heart extracts from various stages of hibernation bouts (upper panel), and their quantitative analyses (lower panel). ACR, active in cold room; Ent, entrance; E-hib, early torpor; L-hib, late torpor; AR, arousal; IB, interbout. Quantitation data represent the mean ± SD of at least four different kidney or heart samples from each stage of hibernation bout. The statistical analyses were shown relative to the ACR group. **p* < 0.05, ***p* < 0.01, ****p* < 0.001.

FIGURE S2 Akt isoforms (Akt1 and Akt2) are differentially expressed in the kidney of 13-lined ground squirrels during hibernation bout. **(A)** Representative immunoblots of Akt isoforms, Akt substrates (GSK3β, PRAS40), and their phosphorylated forms in the squirrel kidney during hibernation bout. **(B)** Quantitative analyses of these protein expressions. **(a)** Akt1 and Akt2; **(b)** the ratio of Akt1/Akt2; **(c)** p-Akt (S473); **(d)** p-Akt (T308); **(e)** total (t-) and phosphor (p)-GSK3β; **(f)** total- (t-) and phosphor-(p)-PRAS40. ACR, active in cold room; Ent, entrance; E-hib, early torpor; L-hib, late torpor; AR, arousal; IB, interbout. Quantitation data represent the mean ± SD of at least four different kidney samples from each stage of hibernation bout. The statistical analyses were shown relative to the ACR group. **p* < 0.05, ****p* < 0.001, *****p* < 0.0001.
