# Supplementary figures and images for: Akt Protein Kinase, miR-200/miR-182 Expression and Epithelial-Mesenchymal Transition Proteins in Hibernating Ground Squirrels
Source: Front Mol Neurosci. 2018 Jan 30;11:22. doi: 10.3389/fnmol.2018.00022 (PMC5797618; doi:10.3389/fnmol.2018.00022)

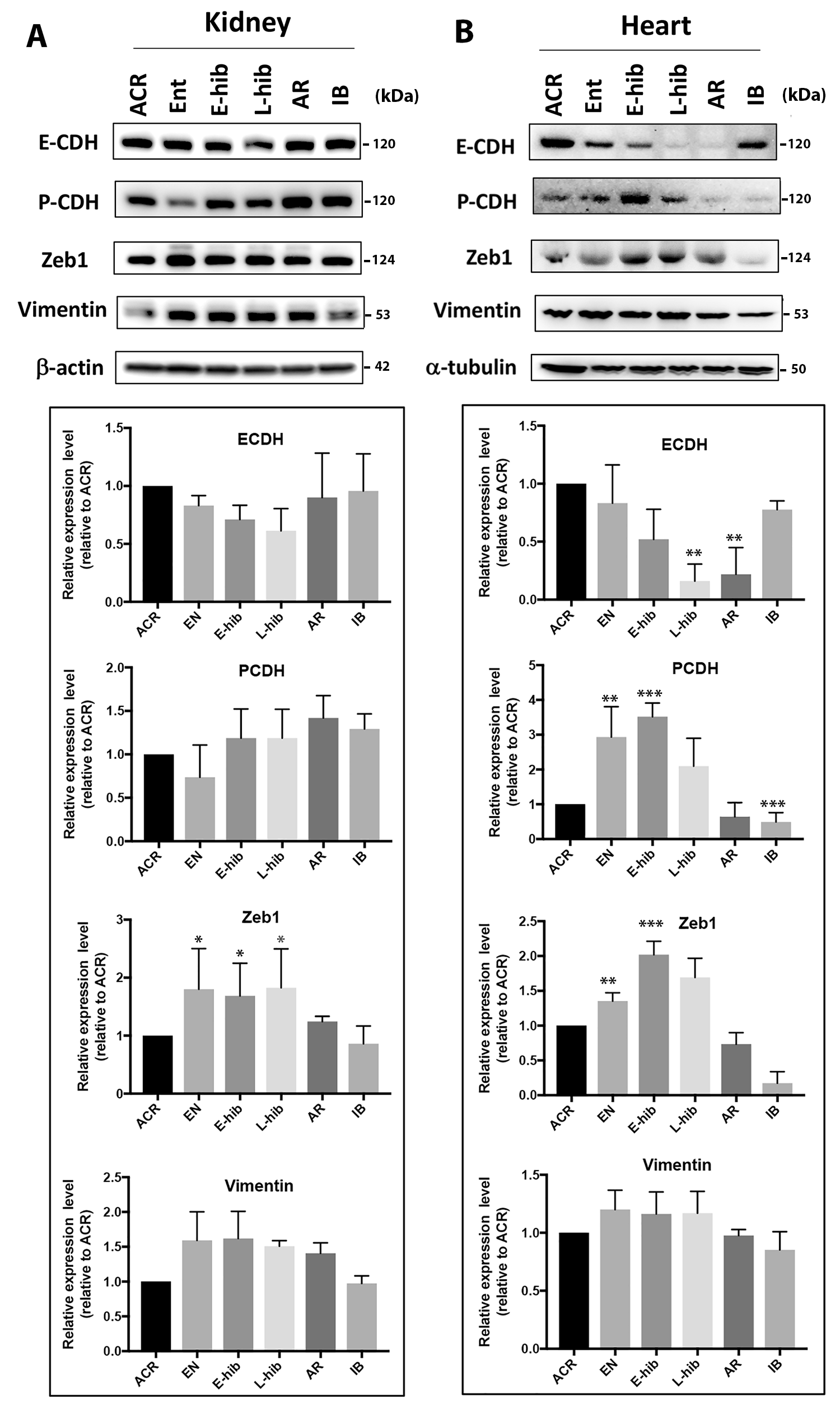

Supplement: FIGURE S1 — Expression levels of EMT-related proteins in the squirrels’ kidney and heart during hibernation bouts. (A) Representative immunoblots of EMT markers in the kidney extracts from various stages of hibernation bouts (upper panel), and their quantitative analyses (lower panel). (B) Representative immunoblots of EMT markers in heart extracts from various stages of hibernation bouts (upper panel), and their quantitative analyses (lower panel). ACR, active in cold room; Ent, entrance; E-hib, early torpor; L-hib, late torpor; AR, arousal; IB, interbout. Quantitation data represent the mean ± SD of at least four different kidney or heart samples from each stage of hibernation bout. The statistical analyses were shown relative to the ACR group. *p < 0.05, **p < 0.01, ***p < 0.001. [file Image_1.tif]

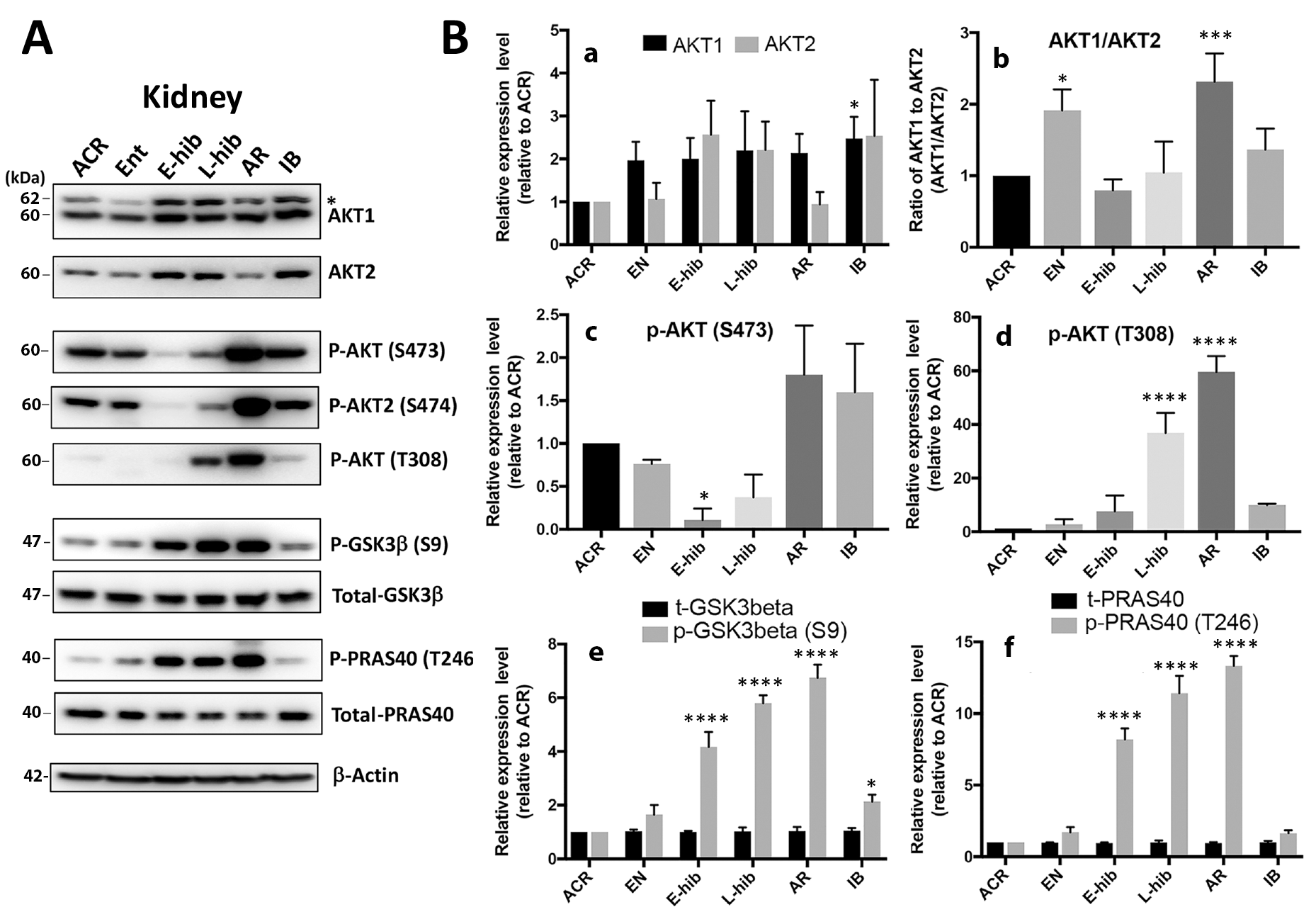

Supplement: FIGURE S2 — Akt isoforms (Akt1 and Akt2) are differentially expressed in the kidney of 13-lined ground squirrels during hibernation bout. (A) Representative immunoblots of Akt isoforms, Akt substrates (GSK3β, PRAS40), and their phosphorylated forms in the squirrel kidney during hibernation bout. (B) Quantitative analyses of these protein expressions. (a) Akt1 and Akt2; (b) the ratio of Akt1/Akt2; (c) p-Akt (S473); (d) p-Akt (T308); (e) total (t-) and phosphor (p)-GSK3β; (f) total- (t-) and phosphor-(p)-PRAS40. ACR, active in cold room; Ent, entrance; E-hib, early torpor; L-hib, late torpor; AR, arousal; IB, interbout. Quantitation data represent the mean ± SD of at least four different kidney samples from each stage of hibernation bout. The statistical analyses were shown relative to the ACR group. *p < 0.05, ***p < 0.001, ****p < 0.0001. [file Image_2.tif]
